# Physical Exercise For Parkinson’s Disease: Clinical And Experimental Evidence

**DOI:** 10.2174/1745017901814010089

**Published:** 2018-03-30

**Authors:** Alessandro Oliveira de Carvalho, Alberto Souza Sá Filho, Eric Murillo-Rodriguez, Nuno Barbosa Rocha, Mauro Giovanni Carta, Sergio Machado

**Affiliations:** 1 Institute of Psichiatry, Federal University of Rio de Janeiro (IPUB/UFRJ), Rio de Janeiro, Brazil; 2 Castelo Branco University, Rio de Janeiro, Brazil;; 3 Physical Activity Neuroscience, Physical Activity Sciences Postgraduate Program - Salgado de Oliveira University, Niterói, Brazil;; 4 Physical Education Department, Faculty of Unidas de Campinas (FacUNICAMPS), Goiânia, GO, Brazil; 5 Politechnique Institute of Porto, Healthy School, Porto, Portugal; 6 Laboratorio de Neurociencias Moleculares e Integrativas Escuela de Medicina, División Ciencias de la Salud Universidad Anáhuac Mayab, Yucatán, Mexico; 7 Laboratory of Panic and Respiration, Institute of Psichiatry, Federal University of Rio de Janeiro (IPUB/UFRJ), Rio de Janeiro, Brazil; 8 Intercontinental Neuroscience Research Group, Yucatán, Mexico

**Keywords:** Parkinson’s disease, Aerobic exercise, Strength training, Brain-derived neurotrophic factor, Physical fitness, Mood impairment

## Abstract

**Background:**

National projections about the increase in the elderly population over 60 years bring with it an increase in the number of people affected by Parkinson's Disease (PD), making it an important public health problem. Therefore, the establishment of effective strategies for intervention in people with PD needs to be more clearly investigated.

**Objective:**

The study aimed to report the effectiveness of exercise on functional capacity and neurobiological mechanisms in people with PD.

**Methods:**

This study is a critical review of the literature.

**Results:**

The progressive death of dopaminergic neurons in the substantia nigra is described as one of the main physiological mechanisms manifested before PD, directly interfering with motor behavior. However, PD is not only related to motor symptoms, but also to cognitive, autonomic, and mood impairments. Such effects may be attenuated by pharmacological influence, but also evidence suggests that the implementation of regular physical exercise programs may exhibit potential benefits over PD. The synthesis and expression of monoaminergic neurotransmitters can act positively on motor disorders, as well as directly or indirectly influence the neuronal plasticity of the brain, restoring neuronal pathways previously affected.

**Conclusion:**

Physical exercise contributes effectively to the treatment of PD, and can play a preventive and maintenance role of physical fitness and mental health.

## 1 INTRODUCTION

Parkinson's Disease (PD) is the second most common prevalent neurodegenerative disease, only behind Alzheimer's disease [1, 2]. Described in 1817 by a physician named James Parkinson [3], it is one of the chronic diseases associated with aging, as well as risk factors such as exposure to drugs, head trauma, pesticides (rotenone, paraquat, maneb) and drugs (cocaine, heroin) [4]. There is also evidence for the mutation of some genes with functions associated with neurotransmitter expression, maintenance and neuronal survival as α-Synuclein (SNCA) and Leucine-Rich Repeat Kinase 2 (LRRK2) (dominant autosomal inheritance), and Parkin (PARK2), PINK1 and DJ-1 (PARK7) among others with autosomal recessive inheritance characteristics [5].

The disability produced by PD makes an important impact on the patient's life and his/her family. The disease progression leads to an increase in the inability to perform the Activities of Daily Living (ADL), loss of independence and a decrease in the quality of life (QoL), besides generating occupational and socioeconomic impairments [6]. According to the World Health Organization, the global incidence rate of PD is estimated to be around 4.5 to 19 per 100,000 inhabitants, affecting both genders and races [7]. Considering the increase in the life expectancy of the world population, and consequently of the aging, it is possible to estimate an increase in the number of people affected by PD, making it an important global public health problem.

The literature suggests that the effects of PD can be attenuated by pharmacological treatment. However, it seems that the drugs available for treatment only provide relief of the symptoms, and do not stop the progression of the disease [8]. Therefore, searching for non-pharmacological treatments to help patients is valid and necessary to bring effective treatment to them.

Within this context, the implementation of regular physical exercise programs, seems to be a viable alternative to demonstrate benefits on motor and non-motor symptoms of PD. In this case, the synthesis and expression of exercise-induced monoaminergic neurotransmitters may explain the significant influence of exercise on neuromotor and mood disorders [9-12]. For example, in the animal model, the synthesis of Dihydroxyphenylacetic Acid (DOPAC) and Homovanallic Acid (HVA), with metabolites associated with dopaminergic levels in the brain, is strongly related to exercise intensity, as well as extracellular levels of dopamine after seven days of training with rats [13]. In humans, the stimulation of a single unilateral motor pattern of flexion and ankle extension is responsible for the dopaminergic release in the contralateral dorsal striatum in people with PD [14]. In addition, the level of activation of the striatal region due to dopamine release may be stimulated to increase upon acquisition of new motor patterns compared to the achievement of the same repeated pattern of movement [15], suggesting that the learning of new movements in some way may contribute to maintenance or increase of dopaminergic levels from simple musculoskeletal contraction.

It is also argued that exercise contributes positively to non-motor deficits (behavior, mood, cognition) in relation to PD. Expression of neurotrophic substances, in particular, BDNF (Brain-Derived Neurotrophic Factor), growth factor promoting proliferation, survival, and maturation of new neurons [16, 17], appear to be induced from one or more physical exercise sessions [18-20]. Such substance is related to increased dendritic arborization and volumes in cortical areas, mitochondrial biogenesis from stimulation of the expression of the PGC-1α transcription factor (peroxisome proliferator-activated receptor gamma coactivator 1 alpha) [21], as well as, it has an an essential role of co-regulating the serotoninergic system [16]. Therefore, these and other neurotrophic factors exhibit a broad plasticity and resilience function on the negative spectrum of the disease [22], and can be modified with an adequate dose of physical exercise.

Despite the preliminary understanding of PD outcomes, a variety of methods and the poor quality of the proposed studies of interventions with exercise [23-25] question their real effectiveness. Thus, some questions about the relationship modality *vs.* dose *vs.* response to the clinical application still need to be better positioned. Therefore, we aimed to report the effectiveness of the intervention through exercise on physical, physiological, and psychological symptoms in people with PD.

## 2 METHODS

This article is a critical narrative review. A literature search was conducted using the databases PubMed, ISI Web of Knowledge, PsycInfo, and Google, using the following terms and their combinations: *“aerobic exercise”, “Parkinson disease”, “mechanism”, “pathophysiology”, “strength training”, “brain-derived neurotrophic factor”, “cognitive function”, “dopamine”*. All articles were published between 1950 and 2017 and in English. Additional references were identified through hand search of the possessed articles. Due to the few randomized clinical trials on the issue, we decided to select any study, *i.e.*, open and controlled studies, case reports, and cohort and observational studies.

### 2.1 Pathophysiology of Parkinson’s Disease

**Fig. (1):**
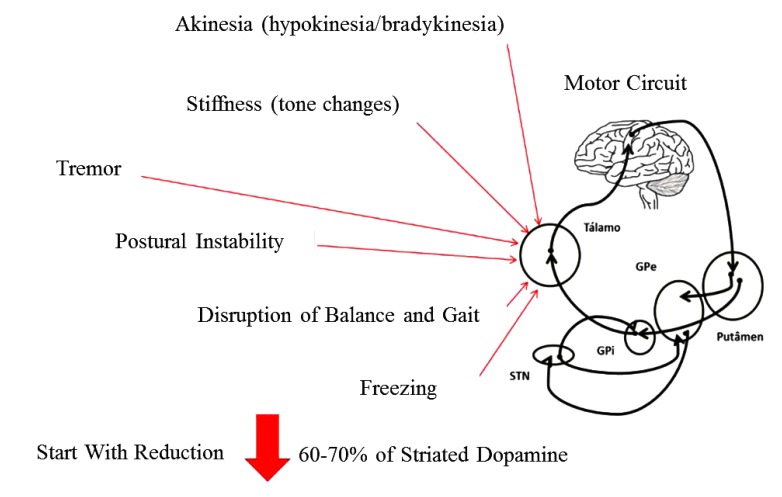
Examples of experimental tests showing a high (a) and a low (b) data dispersion.

The progressive death of dopaminergic neurons in the Substantia Nigra pars compacta (SNpc) located in the midbrain, more precisely in the Basal Ganglia (BG), promotes a significant decrease in the levels of the neurotransmitter dopamine, and as a consequence the functional impairment of the neural circuits (motor, executive and limbic) [26]. The chronic reduction in dopamine levels gives rise to the manifestation of the motor symptoms that characterize this disease. However, the pathophysiology of PD is not limited to the dopaminergic system, that is, neuronal degeneration of other areas of the brain such as the brainstem and cortex competes and even precedes neuronal death in BG. Thus, other neurotransmitter systems (cholinergic, serotonergic and adrenergic) are compromised, thus making PD a multisystemic pathology manifested by a series of motor and non-motor symptoms [2] (Fig. **1**).

The cardinal motor signs of PD are: resting tremor, plastic-type muscular rigidity, bradykinesia (akinesia or hypokinesia), and postural instability. This set of symptoms associated or not, forges characteristic clinical signs in patients with the disease such as gait and balance disorders, mask facies and dysarthria. In addition to these symptoms, a set of sensory (anosmia, pain, paresthesia) autonomic (dysphagia, constipation, urinary incontinence) and cognitive-behavioral symptoms (depression, apathy, dementia) may manifest during the course of the disease [27].

PD has five stages divided according to the impairment caused by the symptoms. The Hoehn & Yahr Scale (H & Y) is the most common clinical instrument used to evaluate the stages of the disease [28]. This scale classifies patients into patients into five stages of disease severity according to signs and symptoms (tremor, stiffness, bradykinesia and postural instability), thus assessing the degree of disability. From stage I to stage III, the patients present mild to moderate disability, and in stages IV and V, severe disability.

### 2.2 Pharmacological Treatment of Parkinson’s Disease

Pharmacological treatment of PD is based on the replacement of dopamine levels in the brain, which generally promotes the temporary improvement of physical disability and control of some of the symptoms. The medication considered gold standard in the treatment of PD motor symptoms is Levodopa (L-dopa), a metabolic precursor of dopamine that crosses the blood-brain barrier easily. Once in the Central Nervous System, L-Dopa is metabolized basally by DOPA decarboxylase, leading to dopamine. L-Dopa is marketed in association with a decarboxylase inhibitor drug (benserazide, carbidopa), preventing the peripheral synthesis of dopamine and ensuring the drug's arrival in the brain [29]. Other drugs are available, in most cases in association with L-dopa, and are divided according to the mechanism of action in order to increase the concentration of dopamine levels such as Monoamine Oxidase-B (MAO-B), Catechol-O-Methyl Transferase (COMT) inhibitors, or dopaminergic agonists such as Pramipexole (Amantadine) [30]. However, pharmacological treatment for PD has limitations ranging from possible drug interactions and side effects (nausea, sleep disorders, hallucinations), to decrease in its efficacy with years of use and the appearance of secondary symptoms such as dyskinesias and fluctuations (wearing off), due to the lack of effectiveness of the treatment of non-motor symptoms of the disease [27, 31]. The choice of drug or association that will be administered to the patient depends on factors such as age, stage of the disease, type of activity performed by the patient, and their mental state.

### 2.3 Effects of Physical Exercise on the Treatment of Parkinson’s Disease

Other therapeutic strategies have been evaluated clinically and scientifically in recent years in the search for an action to reduce clinical problems of PD, such as, non-pharmacological interventions like physiotherapy and physical exercise [32, 33]. Rehabilitation through physical therapy has a variety of goals and methods that generally promote benefits in parkinsonian mobility, posture, and balance. However, some limitations have been observed in a consensual way by some researchers in two topics: in relation to the benefits that seem to be more immediate (acute), and the variety and low methodological quality of the studies [23-25]. Other nonpharmacological approaches to rehabilitation in Parkinson's disease are the practice of different modalities of physical exercises such as walking, running, strength training, whole body vibration and functional exercises, which are related to the reduction in the risk of falls, decreased motor symptoms, motor performance improvements, balance and gait improvements, positive repercussions in quality of life and executive functions [34-39].

For 50 years, physical exercise has been considered as a form of treatment for PD [40]. Recent literature reviews agree on the benefits of physical exercise in improving functional capacity, gait, balance, and strength in patients with PD [24, 41, 42]. Studies on animal models have shown very promising results on the effect of exercise on PD, especially treadmill aerobic training. Lau *et al.* (2011) examined the effects of treadmill exercise on rats, assessing the the coordination of movements and balance, changes in biomarkers of dopaminergic neurons, mitochondrial function and the activity of neurotrophic factors such as Brain-Derived Neurotrophic Factor (BDNF) in the nigrostriatal system, and their results demonstrated that the neuronal and behavioral recovery produced by exercise in the parkinsonian rats was associated with an improvement in mitochondrial function and an increase in BDNF in the nigrostriatal region of the brain [43]. Other animal model studies point to the hypothesis that physical exercise influences brain plasticity favoring neuroregenerative, neuroadaptive and neuroprotective responses mediated by the release of neurotrophic factors such as the Glial cell line-Derived Neurotrophic Factor (GDNF) and the Vascular Endothelial Growth Factor (VEGF) [43-45], and by the reduction of oxidative stress and the production of Reactive Oxygen Species (ROS) [46]. The expression of these trophic factors is closely related to the activation of certain biomolecular signaling cascades. The expression of BDNF in the hippocampus is generally due to the increase in the activation of β-CaMKII (Calmodulin-dependent Protein Kinase II) promoted by aerobic exercise, whereas the increase of central Insulin-like growth factor 1 (IGF-1) in strength training through the greater activation of the Akt pathway (Protein Kinase B) [47].

In research on humans, the results are more controversial, mainly due to the methodological quality of the studies available in the literature, but no less important. Several reviews show that it is evident the great variability of interventions adopted in the treatment of PD with exercise [30, 41, 48]. Many of these studies combine more than one type of intervention in their treatments, demonstrating favorable results in some of their main outcomes, but making it difficult to interpret the role of each type of intervention in improving patients.

Nevertheless, there are good studies available in the literature on the effect of aerobic exercise and strength training in PD patients. Cakit *et al.* (2007) investigated the effects of eight weeks of aerobic training on a treadmill with load bearing for 30 minutes in the symptoms of PD, balance, speed, and distance walked on treadmill and fear of falls. At the end of the study, the authors presented positive results in the group that underwent the intervention in all variables evaluated in comparison to the control group [49].

Kurtais *et al.* (2008) investigated the effects of six weeks of supervised treadmill walking, three times a week for 40 minutes in patients with mild to moderate PD, and observed significant improvements in lower limb functional parameters such as walking, balance, and agility, and in related parameters, the adaptations promoted by aerobic exercise as increase of peak VO_2_ and caloric expenditure in METs [50].

Other authors suggest that forced aerobic exercise (the patient is led to train at a given intensity by another participant) promotes more benefits than voluntary training [51, 52]. Ridgel *et al.* (2009) compared two groups submitted to aerobic training on the cycle ergometer for one hour, three times a week, for eight weeks. One of the groups pedaled at an intensity between 60% and 80% of maximal heart rate which was called a voluntary exercise (EV), the other group pedaled on a double bicycle, assisted by a trainer who maintained a 30% higher HR intensity group EV. According to the authors, the group that did the forced training had a 35% improvement in the motor symptoms of PD, and the same result was not found in the EV group [51]. Recently Alberts *et al.* (2011) in a study with the same methodology corroborated the results found by Ridgel *et al.* (2009), demonstrating that patients who underwent forced training had an improvement of 41% in stiffness, 38% in tremor and 28% in bradykinesia evaluated by a specific clinical instrument. In this same study, Alberts *et al.* (2011) also demonstrated through magnetic functional resonance (fMRI) that aerobic exercise was able to promote increased activity in cortical and subcortical areas, with responses similar to those observed under the effect of antiparkinsonian medication [52].

Studies on strength training in patients with PD are more recent, only in the last 10 years this type of intervention has been investigated [53]. In general, the health benefits provided by strength training (increased strength and muscle mass, bone mineral density) are useful to the needs of patients with PD, especially with regard to functionality and independence related to daily life. For example, Scandalis *et al.* (2001) investigated the effect of eight weeks of strength training with lower limb emphasis twice weekly in 14 patients with moderate PD. The authors demonstrated that in addition to gait improvement, patients with PD increased strength similar to the control group composed of volunteers without the disease [54]. Dibble *et al.* (2006) demonstrated that patients with PD submitted to 12 weeks of high-intensity strength training had increased muscle strength and volume, as well as improvements in mobility [55]. Although in clinical practice, strength training is related to a possible worsening of PD symptoms, as can be observed, there is no evidence in the literature for this association [53].

Few studies have evaluated the effects of physical exercise on non-motor symptoms of PD. Tanaka *et al.* (2008) investigated the effect of combined exercise training (strength, balance, and coordination) on 10 patients with mild to moderate PD in executive functions, and found improvements in focused attention and inhibitory control [38]. Ridgel *et al.* (2011) observed an improvement in the executive functions of patients with PD assessed by the Trail-Making Test (TMT) A and B acutely, that is, immediately after two groups pedal for 40 minutes on a voluntary or forced basis [56]. Physical activity through active video games (Exergames), looks like a potentially useful tool in the field of neurorehabilitation able to offer motor and cognitive improvements in people with neurodegenerative diseases, including PD [57].

### 2.4 Exercise Prescription Based on Evidence for Parkinson’s Disease

It is possible to assume that patients with Parkinson's disease should benefit in the majority of cases with different strategies, which should be prescribed based on a careful clinical evaluation, functional capacity, mental health and cardiorespiratory function. With these data in hand, the physical education or physiotherapy professional will be able to choose the type of training, duration, intensity and other variables to be worked out in order to promote the benefits of exercise to the patients.

The American College of Sports Medicine (ACSM) has published recommendations for the prescription of exercises for parkinsonians [58]. These recommendations are a good guide on what exercises to prescribe for this population and how to do it. One of the key information in this guide is that exercise recommendations for adult health fitness can be applied to parkinsonians, with caveats to the condition and physical limitations that the person presents. Adults with Parkinson's disease may present improvements similar to those of healthy adults in the variables of physical fitness (strength, muscular and aerobic resistance, balance and flexibility) [54], with direct impact on improving functional capacity (Board **1**).

Most of these recommendations apply to those patients who are between the early stage (stage 1 of the Hoehn & Yahr scale) to moderate (stage 3). In stage 4, the patient usually needs ancillary devices for balance and gait, which requires an adaptation of the exercises to the presented physical limitations. While in stage 5, the patient is usually bedridden and with many limitations, requires a palliative approach with emphasis on the prevention of deformities.

## CONCLUSION

Physical exercise contributes to the treatment of Parkinson's disease, and can play a preventive role and maintain physical fitness and health. Its practice must be spread among experts and lay people in order to demystify possible misconceptions about the capacity of a person with physical or functional limitations in performing this type of training. Evidence-based physical exercise and planning geared to the needs and capabilities of this population will produce intangible benefits to parkinsonians. There is a need for greater evidence on doseresponse, the effect of training variables (intensity/ duration/frequency), type of exercise, adherence to treatment and mechanisms involved in each motor intervention, but this does not prevent that physical exercise is considered essential in the effect the treatment of Parkinson's disease.

## Figures and Tables

**Board 1:** Recommendations for prescription of training in Parkinson's disease.

AEROBIC	
Frequency:	20-60 min/day - 3-5 days/week
Intensity:	Light (< 40% HRR or VO2R); Moderate (40-60% HRR or VO2R); Hard (> 60% HRR or VO2R)
Type:	Walk or Cicle
RESISTANCE	
Frequency:	2-3x/week
Intensity:	Light (40-50% 1MR); Moderate (60-80% 1MR); Hard (>80% 1MR)
Type:	Progressive (2-4 sets; 8-15 reps; Large Muscle Groups)
FLEXIBILITY	
Frequency:	2-3x/week
Intensity:	10-30 sec (to the point of discomfort)
Type:	Static Stretching, Dynamic, and PNF, with Emphasis on Spine and Trunk
BALANCE	
Frequency:	10-15 min - 2-3x/week
Intensity:	There is no Evidence of the Intensity
Type:	Exercises Involving Motor Skills (Balance, Agility, Coordination, Gait, and Proprioception)

## LIST OF ABBREVIATIONS

PD: = Parkinson’s Disease
SNCA: = α-Synclein
LRRK2: = Leucine-Rich Repeat Kinase
DOPAC: = Dihydroxyphenylacetic Acid
DOPAC: = Dihydroxyphenylacetic Acid
HVA: = Homovanylic Acid
PD: = PGC 1 α Peroxisome proliferator activated receptor gama coativator 1 alpha
BG: = Basal Ganglia
L-DOPA: = Levodopa
COMT: = Cathecol-O-Methyl Transferase
BDNF: = Brain Derived Neurotrophic Factor
GDNF: = Glial Cell Derived Neurotrophic Factor
VEGF: = Vascular Endothelial Growth Factor
ROS: = Reactive Oxygen Species
IGF 1: = Insulin-Like Growth Factor 1
fMRI: = Functional Magnetic Resonance
TMT: = Trail Making Test
ACSM: = American College of Sports Medicine
